# The Use of Artificial Intelligence in Assessing Affective States in Livestock

**DOI:** 10.3389/fvets.2021.715261

**Published:** 2021-08-02

**Authors:** Suresh Neethirajan

**Affiliations:** Farmworx, Animal Sciences Department, Wageningen University & Research, Wageningen, Netherlands

**Keywords:** animal emotions, animal welfare, sensors, affective states, emotion modeling, animal-based measures, artificial intelligence

## Abstract

In order to promote the welfare of farm animals, there is a need to be able to recognize, register and monitor their affective states. Numerous studies show that just like humans, non-human animals are able to feel pain, fear and joy amongst other emotions, too. While behaviorally testing individual animals to identify positive or negative states is a time and labor consuming task to complete, artificial intelligence and machine learning open up a whole new field of science to automatize emotion recognition in production animals. By using sensors and monitoring indirect measures of changes in affective states, self-learning computational mechanisms will allow an effective categorization of emotions and consequently can help farmers to respond accordingly. Not only will this possibility be an efficient method to improve animal welfare, but early detection of stress and fear can also improve productivity and reduce the need for veterinary assistance on the farm. Whereas affective computing in human research has received increasing attention, the knowledge gained on human emotions is yet to be applied to non-human animals. Therefore, a multidisciplinary approach should be taken to combine fields such as affective computing, bioengineering and applied ethology in order to address the current theoretical and practical obstacles that are yet to be overcome.

## Introduction

Compiling evidence that non-human animals are able to use complex cognitive process and show emotions is appearing. An increasing body of literature has investigated the complexity of the animal mind and their ability to show emotions such as fear, pleasure, jealousy and grief (1–4). This evidence leads us to the question of how we could recognize and monitor affective states in livestock. This knowledge is essential to improve the welfare of farm animals kept in captivity through recognizing negative emotions and acting upon them, while promoting positive affective states. However, understanding the animal mind and their emotions is complex as we will only ever be able to use indirect measures to infer affective states. Experimentally testing animal emotions requires careful experimental set up and interpretation of data, which is commonly done through elaborate behavioural observations and tests (judgement bias, attention bias, spatial judgement task etc.,) which is both labour and time intensive, and unfeasible to be practiced on a large scale. In addition, the behavioural testing requires extensive animal training, while some individuals may be unable to be trained which then excludes a certain proportion of animals from being assessed. Artificial intelligence, and in particular machine learning and complex algorithms, combined with the frequent collection of indirect measures with sensors that reflect affective states, can provide us with an objective, efficient and automated means of monitoring animal welfare. While affective computing has received more attention in humans due to its widespread implications for e.g. neuromarketing (5), entertainment (5), monitoring mental health (6, 7) and human-robot interactions (8), the application of affective computing on farm animal welfare is however in its infancy and therefore, more multidisciplinary and exploratory studies are needed to further develop this highly promising field.

### The Importance of Understanding the Animal Mind

Understanding when an animal is in distress, pain or fear is essential to improve animal welfare and allow the most animal-friendly practices within farms. Additionally, and maybe even just as important; animal welfare can only really be optimized once we get a better understanding of what promotes positive emotions. We therefore need to understand what types of environments and practices promote positive affective states and what factors can cause stress and discomfort. Moreover, effective recognition of negative affective states and subsequently decreasing stress by removing the causal factors may increase animal productivity, reduce disease and prevent infection (9–15).

### Emotions and Affective States

Before we can understand how to effectively measure emotions in non-human animals, a definition of the concept is required and an understanding of how emotion differs from affective state and mood. An affective state encompasses both emotions, which are short-term and directed at a particular source, and mood, which is less intense than an emotion and can appear without a particular stimulus to cause it (16). Most definitions of emotion originated from the field of psychology, but no consensus has been achieved yet on the concept, nor the number of emotions that exist. One definition that is relevant when we are looking for methods to measure emotion, is the definition by Broom and Johnson which states that an emotion is “a physiologically describable component of a feeling characterized by electrical and neurochemical activity in particular regions of the brain, autonomic nervous system activity, hormone release and peripheral consequences including behaviour”, whereas a feeling also involves the perceptual awareness of this emotion by the individual (17). Affective states are important for welfare because of their subjective “feeling” component, i.e. because they are experienced as pleasant or unpleasant. If there is an emotion without perceptual awareness, then such an emotion may be of no relevance to animal welfare. Izard has proposed emotion schemas as an attempt to scientifically define emotions (18). Emotion schemas consist of “emotion interacting dynamically with cognitive and perceptual processes to influence behaviour and mind” (19). An internal or external stimulus can cause a particular emotion, accompanied by measurable changes that in turn, can lead to a behavioural response to cope with the challenges of the physical and social environment.

Paul Eckman suggested six basic emotions: happiness, sadness, fear, disgust, anger and surprise (20). The criteria that he used to define a basic emotion consist of distinctive facial expressions that are universal, the presence in other primates, a distinctive psychology, a rapid onset, brief duration and that the appraisal of them is automatic (21). Additionally, Robert Plutchik also suggested that emotions should provide a function that aids survival and therefore touched upon the evolutionary significance of emotion (22). Whereas fear is the emotion that has received the greatest attention, compiling evidence suggests that other emotions such as grief, jealousy and happiness are apparent in non-human animals, too (1–4, 23). Even though measuring discrete emotions is an exciting and relatively unexplored field, more commonly a dimensional approach is taken to measure emotion in non-human animals. The main two dimensions measured here are the level of arousal (high/low) and emotional valence (positive vs. negative depending on the level of reward/punishment of the stimulus) (16). Authors such as Ekman and Plutchik have not considered sensory and homeostatic states such as pain, hunger, nausea etc. in the definition of emotion. However, according to Jaak Panksepp, these homeostatic affective states rely on different brain mechanisms and should be considered as a separate category from 'Ekmanian' emotions caused by external events (18). An attempt to create a common definition of emotion to the affective sciences is a fruitless debate (24) due to the differences in disciplines such as psychology, neuroscience, philosophy and animal sciences. Few theories consider emotions as concomitants of both bodily and cognitive elements (25). Due to the reciprocal influence between brain and body systems such as nervous system and immune system, the physiological account of stress can be illustrated as an emotional condition (25). Although stress can be considered as an affective state, it rather fits the category of long-term affective states rather than the category of emotions which are short-term states caused by specific stimuli. The scope of the paper is about affective states (i.e. states with a valenced feeling element) and that this broad category includes sensory and homeostatic affective states, emotions, and moods.

### How Can We Measure Affective States in Non-Human Animals?

However, one major obstacle within the field of animal affective states is our inability to accurately measure affective states. Whereas the identification and recognition of affective states in humans is commonly accompanied by questionnaires to validate the effectiveness of the methods in question, getting a confirmation in the same method as humans is impossible. Research on human affective states has focused on the ability to measure emotion based on verbal speech, written text and hand gestures, amongst other measures, which are all means of communication that are not or less used by non-human animals. A great number of behavioural tests have been employed to allow assessment of affective states in other non-human animals, including open field, human interaction, cognitive bias and elevated plus maze tests (26–29). More studies keep appearing that show a correlation between indirect measures and the results of such behavioural tests, and therefore such measures provide a more efficient method of monitoring animal affective state without the need for individual behavioural testing. Such indirect measures include hormonal assessments, physiological measures, facial expression, brain activity, thermal imaging, vocalisations and movement (30). In multiple farm animal species (cattle, sheep, goats, horses, pigs, poultry), emotional valency has been associated with particular vocalisations (31–37), changes in nasal temperature (38) or eye temperature (39–41), cortisol levels (39, 42–44), heart rate and heart rate variability (40, 45–49), respiration rate (50) but also facial expression and the (change in) position of the ears and tail (31, 43, 46, 51–62). See Table 1 for a collection of studies that tested these measures and how they relate to emotional valence. The papers cited in the table were collected using the Web of Science tool. Past 10-year peer reviewed published papers were only considered for inclusion in the table. Keywords for searching in the web of science database included farm animals, pigs, cows, horses, sheep, chickens, emotions, indicators of welfare, and affective states. Boolean and individual searches using the above-mentioned keywords were used for the literature collection.

**Table 1:** Summary of literature that measures indicators of affective states using unimodal and multimodal approaches in farm animals.

**Type of measure**	**Indicator**	**Affective state**	**Species**	**Stimulus**	**Method for measurement**	**Reference**
**Unimodal approach**						
Behaviour, posture, hormonal	Play, vocalisations, tail movements, defecating, urinating, tail and ear position, salivary cortisol	Positive and negative valence	Pigs	Rewarding or aversive event	Behavioural observations	Du et al. (32)
Hormonal	Salivary oxytocin	Positive emotions	Pigs	Different times during lactation, after farrowing	Novel competition assay based on AlphaLISA	Battini et al. (63)
Hormonal	Salivary oxytocin, behaviours	Positive human-animal interactions	Pigs, cattle, goats	Stroking by familiar and unfamiliar humans	ELISA, behavioural observations	Von Borell et al. (44)
Temperature	Head, eye and comb surface temperatures	Stress	Hens	Handling, holding in box	Infrared thermography	Peeters et al. (41)
Posture	Eye white, ear posture	Level of excitement	Dairy cows	Avoidance distance test, housing	Behavioural observations	Gaughan et al. (64)
Posture	Tail and ear postures	Positive emotions	Pigs	Different habitats	Behavioural observation	Mota-Rojas et al. (61)
Posture	Facial expression	Pain	Sheep	Pain	Automatic pipeline for facial feature detection and pain classification	Oliveira et al. (57), Czycholl et al. (60)
Posture	Facial expression	Pain	Sheep	Footrot, mastitis	Manual scoring with sheep pain facial expression scale	Lu et al. (56)
Posture	Facial expressions	Pain	Pigs	Tail-cropping, castration	Piglet grimace scale	Gómez et al. (62)
Posture	Ear posture: upright, forward, backward, hanging	Low arousal positive emotional state	Dairy cow	Stroking	Focal observations	Proctor and Carder (52)
Posture	Facial expression	Pain	Lamb	Before and after tail docking	Manual scoring based on the lamb grimace scale	Hintze et al. (54)
Physiological	Respiration rate	Stress	Cattle	Heat stress	Manual observation of flank movement	Tarantola et al. (65)
**Multimodal Approach**						
Physiological	Haematological and biochemical parameters, hair cortisol	Fear, general welfare	Beef cattle	Different housing conditions	Automated haematology analyser, spectrophotometric analysis, high-performance liquid chromatography	Cihan et al. (66)
Physiological, behaviour	Heart rate, stepping, tail flicking, kicking	Stress	Dairy cows	Veterinary procedure, subjects priorly exposed to stroking or human presence	Behavioural observations, commercial heart rate monitoring system	Mott et al. (47)
Posture	Eye wrinkles, eyelid shape, presence of eye white, angle between eyeball and highest wrinkle	Positive and negative emotional state	Horses	Positive (grooming, food anticipation) and negative (food competition, plastic bag) condition	coreIDRAW Graphics Suite X7, manual observations	McLennan et al. (55)
Posture	Eye white visibility, behaviours including vocalisations, aggressive behaviours, positive behaviours	Positive emotional state	Dairy cows	Stroking	Behavioural observations, manual eye white measurement from videos	Guesgen et al. (53)
Posture, heart rate	Heart rate, tail-swishing, head-tossing, bucks	Discomfort, stress, fear	Horses	Riding in Rollkur (coercively hyperflexion of neck)	Preference test, fear test and behavioural observations	Schmied et al. (46)
Posture, hormonal, physiological	Facial expression, oxytocin, cortisol, heart rate, heart rate variability	Positive emotion	Horses	Tactile stimulation	Behavioural observation, ELISA, radio immunology, felt for heart monitoring	Lürzel et al. (43)
Temperature	Nasal temperature	Positive and negative valence	Dairy cows	Stroking	Behavioural observations, infrared thermometer gun	Valera et al. (38)
Temperature, hormonal	Maximum eye temperature, salivary cortisol	Stress	Horses	Before and after jumping competition	Infrared thermography, immunoassay	Bartolomé et al. (39)
Temperature, physiology	Eye temperature, heart rate	Stress	Horses	Before and after jumping competition	Portable infrared thermography, portable pulsometer	Edgar et al. (40)
Temperature, physiology	Respiration rate, temperature	Stress	Calves	No particular stressor, correlated the two	Infrared thermography, respiration rate	Reefmann et al. (50)

### The Need for a Multimodal Approach

These measures all have their advantages and disadvantages. Ideally, the measure that is used as an indicator of affective state will be (1) non-invasive, (2) will produce high-quality data with low sensitivity to environmental disturbances, (3) can be automated to reduce the time and labour required to collect the data, (4) is able to track affective states of individual animals, (5) is not too costly and (6) can identify subtle changes to allow prevention of negative affective state. Naturally, no measure to date is able to check all these boxes, and therefore the main emphasis is on defining a reliable indicator that is consistent across different contexts. One issue with using just a single measure, however, is that a positive and negative emotional valence might have a similar effect on the measure used (38). Furthermore, measures are not always consistent between different studies (67). This highlights the importance of implementing a multimodal approach, combining different measures to get a better understanding of the affective state of the animals. Such an approach has already been emphasized within human affective research (68, 69) as multimodal approaches are able to assess affective states more accurately than any single measure could do on its own. Affective expression consists of complex coordination of signals made of physiology; facial expressions; posture and behavioural indicators. Multimodality signals consists of various channels of expressions such as audio, postures, gestures/activity, visual and eye gage etc. Unimodal signals have the drawback because it is associated with only one type of expression (70). Unimodal affect detectors are also inherently noisy due to the association between affective states and the specific individual signal. The coupling between observable expressions and experience of specific affective states is weaker for unimodal in comparison to the multimodal approach (70). For example: Interactions of auditory and visual emotional information (multi-cues) are not only limited to communication aspects but also to contexts such as environmental cues, contagion aspects and social interactions. Current advancements in human research are moving from traditional unimodal to multimodal approaches in assessing affective states due to the need for enhanced accuracy and the ability to integrate multiple sensory channels. To enable the welfare monitoring and decision support systems in accurately interpreting the affective states of farm animals, multimodal based affective computing is an absolute need. In order to create a reliable framework incorporating multiple factors, the different measures have to be temporally synchronized, and an in-depth knowledge needs to be available about how the measures intercorrelate. The most important limiting factor here is that a large amount of reproducible data needs to be collected to build such a framework.

### Self-Learning Algorithms Are the Answer

Artificial intelligence and machine learning can provide an efficient, objective and automatic self-learning mechanism that could classify different affective states based on multiple factors, while using a sensor-based approach to frequently collect data (30, 71–75). Machine learning consists of computer algorithms learning as more data are added to the system, while being able to recognize patterns and/or features and categorize them. Such systems are able to analyse and predict affective states and notify the user of any abnormalities. So, instead of manually analysing video or photo footage, or live observations of animals by often multiple experimenters recording different measures, using sensors will reduce the amount of time and labour required to collect needed data. To allow the system to recognize affective changes, a baseline level of the measures is required to understand animals in a “neutral” environment, and through experimentally testing the animals in a known positive or negative environment, inferences can then be made about affective states changes. Machine learning mechanisms require a large amount of high-quality data which are used to train the model. By using sensors that can continuously collect data, subtle changes are easier to pick up and simultaneously, large amounts of data continue to be collected that can feed the algorithm and improve its accuracy. As more data are collected, the accuracy of subtle changes will improve too, which would allow early detection and therefore prevention of e.g. tension within the group that has the potential to escalate, disease or infection, and other health problems. Such data would have to be collected for each species in investigation, and an interdisciplinary approach is needed combining computational science, animal science and ethology.

Currently, research within affective computing for non-human animals is pioneering. One of the first steps to take is choosing measures that complement each other and collectively give an accurate representation of the animal's affective state. Promising sensors include infrared thermography and facial expression / posture. What sets apart these types of sensors is their ability to recognize individual animals while recognizing and investigating e.g. the temperature of a particular regions of interest, such as the eyes, ears or tails. Moreover, it has already been shown that the equipment needed to collect such non-invasive data can be mobilized, increasing the feasibility to employ such equipment within a larger industrial setting (40, 50, 76). More and more studies demonstrate the applications of automated systems within a farm setting, highlighting the potential of this field, ranging from surveying pig enclosure usage to identifying heat stress (77) to infrared thermography to track cattle health (76). Human affective research is only just starting to understand the importance of multimodal approaches, and this knowledge should be used to develop models for non-human animals, too. Research on humans have resulted in complex systems that allow accurate sentiment analysis, preference detection (5) using qualitative and quantitative data such as facial expressions, body gestures, phonetic and acoustic properties of spoken language, word use and grammar in written text and more (8, 68). Different algorithms have been designed using computational methodologies such as hidden Markov chains, Bayesian networks and Gaussian mixture models to e.g. recognize facial expressions (78) and allow automatic detection of regions of interest such as the nose (79). An end-to-end emotion prediction task using a multimodal system from visual and speech data was developed by Tzirakis et al., 2017 (80). The comparative analysis by testing and validation of the emotion prediction system proved that multimodal model is a better valence dimension assessor than the unimodal model. Utilizing the multimodal functional near-infrared spectroscopy (fNIRS), electroencephalograph (EEG) wearable sensors, and capture of facial expressions through video cameras, Sun et al., 2020 (81) investigated the relationship between multimodal brain activity and spontaneous facial affective expressions. The strength of the emotional valence correlation to affective expressions revealed that the multimodal method outperformed the unimodal approaches individually. The outcomes from this study also emphasized the utilization of facial expression and wearable neuroimaging sensors for affective brain-computer applications.

### Limitations and Challenges to Overcome

However, there are multiple theoretical and practical challenges still to overcome. First of all, sensors that provide high-quality multimodal data except for video and thermal infrared cameras are not yet commercially available plus the high costs associated with them might deter farmers to employing such technology. Even though farmers can financially benefit from the early detection of health problems due to a reduction in antibiotic usage and need for veterinary assistance, accompanied by an improvement in welfare of their animals, farmers could be concerned due to data use, misuse and storage and the monetization of data (82). In addition to the financial inability to invest in such technologies, the access to a reliable internet connection and high computing power are essential for the functioning of the algorithms and alerting the farmer timely, which poses a limit to digital farming technologies (74). Most farmers do not have the money to invest in either the research or technology, which means that funding within this field should come from governmental bodies and/or subsidies. Numerous studies have shown that improved welfare in farm animals can positively affect the productivity of the farm and the quality of the animal product. For example, higher levels in serotonin (a hormone associated with positive affective states) can increase calcium levels in dairy cows (14), heat stress in dairy cattle can reduce milk yield and quality (83), stress can reduce meat quality in multiple species (84, 85) and prolonged stress can increase disease risk (12, 13). Ensuring a positive affective state in production animals therefore is not only a benefit to the animals themselves, but also the farmer and consumers. In order for grants to be given, an increase in the public awareness on the complexity of the animal mind, including emotionality, personality, intelligence and cognition should be addressed to facilitate the recognition of the importance of this field.

Other than limitations from the farmer's perspective and funding limitations, the accuracy of the measures is an important topic of discussion as well regarding the practicality of using artificial intelligence for farm animal monitoring. First of all, compiling evidence within ethology and behavioural ecology pinpoints the large inter-individual differences in personality, behaviour and in effect, emotionality (86–89). Such variation will have to be incorporated within the algorithm to avoid creating a detection bias. Secondly, affective computing and applied ethology are two distinct disciplines and a lack of interdisciplinary studies prevents the exchange of valuable information between the fields. An interdisciplinary approach should be taken to combine ethological studies with modern, advanced computing systems and bioengineering approaches to create accurate, efficient, species-specific algorithms fine-tuned to the complexity of the animal's behaviour. Furthermore, even though the field of affective computing in farm animals is rising, many areas remain unexplored. Several sensory systems and means of inter- and intraspecific communication have not been utilized as of yet in order to explore emotionality and emotional contagion between animals. Despite our increasing awareness of the importance of olfaction within animal ecology, such as stress signaling to conspecifics when presented a predator odour (90) or evidence that stress hormones can affect olfactory regulation and perception (91), this area remains unaddressed in the context of affective state recognition. The presence of biomarkers in sweat, such as volatile organic components that are created in stressful environments (92), in combination with scent marks and fecal or urinal excretion of compounds remains an unexplored indicator of affective states. Despite the challenges that olfactory studies bring, such as technical challenges like consistent stimulus delivery, environmental disturbances (wind, other smells, physical factors that affect volatility and spatial distribution), distances to the source of the odour, among others, each dimension that contributes to animal emotionality should be explored, including olfactory factors that might indicate the inner state of the group or individual.

## Concluding Remarks

The Implications and usage of artificial intelligence to monitor farm animal affective state are widespread and rising technologies within human studies should be utilized and adapted to broaden the purposes of affective computing. Despite many obstacles that are yet to be overcome, it is important to recognize the problems and goals that each individual farmer might face, in order to tailor smart systems to the needs of the farm and in effect, its animals. Whereas the need for reducing disease, infection or conflict might be higher in some places and/or species, other farmers might desire to adopt even more animal-friendly and humane practices and want to focus on monitoring positive affective states to uplift animal welfare. All these factors will in turn affect the most efficient and effective technological systems and measures taken to address such goals. Public awareness should be boosted on the presence and importance of animal emotionality so that technology not only advances to identify human cyberbullying, utilize neuromarketing and promote affective entertainment, but also improve the quality of life of the billions of domesticated animals that are born and bred in human captivity.

## Author Contributions

The author confirms being the sole contributor of this work and has approved it for publication.

## Conflict of Interest

The author declares that the research was conducted in the absence of any commercial or financial relationships that could be construed as a potential conflict of interest.

## Publisher's Note

All claims expressed in this article are solely those of the authors and do not necessarily represent those of their affiliated organizations, or those of the publisher, the editors and the reviewers. Any product that may be evaluated in this article, or claim that may be made by its manufacturer, is not guaranteed or endorsed by the publisher.
